# “Basics of AI Prompts for Psychiatrists: From Diagnosis to Treatment”

**DOI:** 10.1192/j.eurpsy.2026.11528

**Published:** 2026-07-31

**Authors:** M. V. Ganduri

**Affiliations:** 1 Psychiatry, Surabhi Institute of Medical Sciences, Siddipet; 2 Psychiatry, MV Clinics, Hyderabad, India

## Abstract

**Introduction:**

Artificial Intelligence (AI) is rapidly reshaping psychiatric practice by offering novel tools for diagnosis, treatment planning, and patient engagement. Psychiatry has traditionally relied on nuanced clinical judgment, but recent advancements in AI prompt systems suggest they may augment clinicians’ cognitive processes and therapeutic interactions. This study aims to introduce psychiatrists to the fundamentals of AI prompt engineering and explore its practical applications in mental healthcare.

**Objectives:**

To introduce psychiatrists to the core principles of AI prompt engineering and its relevance in clinical psychiatry.

To examine how structured AI prompts can assist in diagnostic clarification, treatment planning, and therapeutic interventions.

To evaluate the role of AI as a cognitive support tool that enhances, rather than replaces, clinician judgment and patient interaction.

To demonstrate practical applications of AI prompts through case-based vignettes, including psychoeducation, cognitive restructuring, and motivational interviewing.

To highlight ethical considerations, opportunities, and limitations of incorporating AI prompt systems into psychiatric practice.

**Methods:**

A narrative review of recent literature, complemented by case-based demonstrations from clinical practice, was conducted. Scenarios included diagnostic clarification using DSM5/ICD-11 criteria, integration of pharmacological and psychotherapeutic algorithms, and patient-facing AI interactions for psychoeducation and engagement. Structured AI prompts were tested in simulated vignettes to assess feasibility and clinical relevance.

**Results:**

Preliminary findings indicate that AI prompts can support psychiatrists by reducing cognitive load, streamlining documentation, and improving accuracy in case formulation. AI-assisted tools demonstrated potential in delivering cognitive restructuring, guided mindfulness, and motivational interviewing techniques. Importantly, these systems acted as cognitive extenders rather than replacements, enhancing decision-making and clinician–patient communication.

**Conclusions:**

AI-driven prompts represent an innovative adjunct in psychiatry, bridging diagnostic reasoning, treatment planning, and therapeutic interventions. By equipping psychiatrists with foundational knowledge of prompt-based AI systems, mental health services can become more efficient, scalable, and patient-centered, while maintaining ethical safeguards.

**Disclosure of Interest:**

None Declared

